# Three-hundred cases of Spiegelberg ICP monitoring for hydrocephalus and CSF disorders: the Queen Square experience

**DOI:** 10.1186/2045-8118-12-S1-O14

**Published:** 2015-09-18

**Authors:** Aswin Chari, Edward W Dyson, Andrew R Stevens, Simon D Thompson, Claudia Craven, Samir A Matloob, Huan Wee Chan, Syed N Shah, Tarek Mostafa, Neekhil A Patel, Jinendra Ekanayake, Patricia Haylock-Vize, Ahmed K Toma, Laurence D Watkins

**Affiliations:** 1 Victor Horsley Department of Neurosurgery, National Hospital For Neurology and Neurosurgery, UK

## Introduction

Although invasive intracranial pressure monitoring (ICPM) has been a mainstay in the management of traumatic brain injury for a number of years, its use in an elective or emergency setting to guide management of patients with hydrocephalus and other CSF disorders is a relatively new concept. This single centre experience examines the safety profile and utility of ICPM in this patient population.

## Methods

We retrospectively reviewed all cases of monitoring over a 10 year period at the National Hospital for Neurology and Neurosurgery, London, UK. 338 cases were identified. Case records were reviewed for diagnosis, indication of ICPM, duration, complications and outcome. All operations utilised Spiegelberg ICP monitors, inserted in a protocolised fashion under sedation.

## Results

ICPM was undertaken for a number of different conditions including undiagnosed headache (20.4%), IIH (28.7%), NPH (5.3%), high-pressure hydrocephalus (eg congenital/post-traumatic/post-SAH) (17.2%) and Chiari malformations/syringomyelia (13.6%). Indications for ICPM included headache (74.0%), visual disturbance (6.2%), gait disturbance (6.2%) and cognitive disturbance (5.0%). Mean monitoring time was 37.3 hrs (range 12-154 hrs). Monitoring was conducted in the presence of a CSF shunt (50.6%), venous stent (3.7%) and previous cranial decompression (6.5%). Dynamic monitoring (eg with different shunt settings or pre/post venous stent insertion) was undertaken in 12.4%. Outcomes from ICPM included insertion of new CSF shunt (21.0%), revision of CSF shunt (13.0%), insertion of venous stent (6.5%), insertion of and lumbar drains for infusion studies (3.6%); importantly, non-operative treatment was pursued in a number of cases including shunt valve adjustment (7.7%) and conservative management (29.9%). Complications included superficial infection (4 patients, 1.2%), symptomatic intracerebral haematoma (1 patient, 0.3%) and misplacement (3 patients, 0.9%); importantly, there were no cases of deep intracranial infection and the only case of seizures was in the patient with the intracerebral haematoma.

## Conclusion

This is the largest known series of ICPM for CSF disorders. It shows that ICP monitoring is a safe procedure and may be undertaken as part of routine protocol in the management of complex hydrocephalus patients. The number of cases that were subsequently managed conservatively or with a simple valve adjustment (37.6%) indicates the utility in terms of reducing operative interventions. Further evaluation of positive and negative predictive values based on the results of ICP monitoring and health-economic analyses will push the case for routine ICP monitoring prior to definitive management of all hydrocephalus patients.
